# Isolation of *Alcohol Dehydrogenase* cDNA and Basal Regulatory Region from *Metroxylon sagu*

**DOI:** 10.5402/2012/839427

**Published:** 2012-08-26

**Authors:** Ching Ching Wee, Hairul Azman Roslan

**Affiliations:** Genetic Engineering Laboratory, Department of Molecular Biology, Faculty of Resource Science and Technology, Universiti Malaysia Sarawak, Kota Samarahan, 94300 Sarawak, Malaysia

## Abstract

Alcohol dehydrogenase (Adh) is a versatile enzyme involved in many biochemical pathways in plants such as in germination and stress tolerance. Sago palm is plant with much importance to the state of Sarawak as one of the most important crops that bring revenue with the advantage of being able to withstand various biotic and abiotic stresses such as heat, pathogens, and water logging. Here we report the isolation of sago palm *Adh* cDNA and its putative promoter region via the use of rapid amplification of cDNA ends (RACE) and genomic walking. The isolated cDNA was characterized and determined to be 1464 bp long encoding for 380 amino acids. BLAST analysis showed that the *Adh* is similar to the *Adh*1 group with 91% and 85% homology with *Elaeis guineensis* and *Washingtonia robusta*, respectively. The putative basal *msAdh*1 regulatory region was further determined to contain promoter signals of TATA and AGGA boxes and predicted amino acids analyses showed several *Adh*-specific motifs such as the two zinc-binding domains that bind to the adenosine ribose of the coenzyme and binding to alcohol substrate. A phylogenetic tree was also constructed using the predicted amino acid showed clear separation of *Adh* from bacteria and clustered within the plant *Adh* group.

## 1. Introduction

Alcohol dehydrogenase (Adh) is an enzyme involved in various biological activities such as in the germination and abiotic stresses in plants [1–3]. Previous studies have shown that there are between two or three *Adh* loci in flowering plants with exception in *Arabidopsis *[4, 5]. Previous Adh protein work on sago palm, a flood-tolerant plant, by Roslan et al. [6] detected the presence of Adh in the leaf and roots. A higher Adh enzyme expression was observed in sago palm young shoots compared to the other part of *Metroxylon sagu *[6]. The finding was consistent with those of Padmanabhan and Sahi [7] that reported a greater increase in Adh activity in the leaves than the roots of sunflower that was treated with high phosphorus. In contrast, in flood-intolerant plants such as *Arabidopsis* and pea, increased Adh activity was determined in the roots than in the shoots under anaerobic condition [8, 9]. A higher expression level in different tissue and developmental stage may be because the cells are dividing and exposed to many stresses [10].

The discovery of Adh protein expression in young leaf prompted the work to isolate the *Adh* gene from sago palm. The isolation of the regulatory region was also conducted to further understand the regulation of *Adh* in sago palm. *Adh* gene have been isolated from several techniques from a number of plants such as in *Arabidopsis thaliana, *barley, maize [4, 5, 11], and including *Washingtonia robusta*, a member of same Arecaceae family with *Metroxylon sagu* [12, 13].

In this study, we report the isolation of full length *Adh* cDNA and the regulatory sequences from sago palm leaf using RACE and genomic walking methods. Full length cDNA was isolated using the RACE technique that is faster and less laborious compared to the screening of cDNA library by using gene-specific probe [14]. The *Adh* regulatory sequences of *M. sagu* was also isolated using the genomic walking.

## 2. Material and Methods

### 2.1. Plant Material

Young leaves of sago palm (*Metroxylon sagu*) were obtained from Universiti Malaysia Sarawak (UNIMAS) plant house. The stems of the leaves were discarded. The samples were sterilised with 70% ethanol before being cut into small pieces. The samples were stored at −80°C to preserve the RNA integrity.

### 2.2. Isolation of RNA and DNA

RNA isolation was carried out according to the method described by Wee and Roslan [15] while total genomic DNA was extracted from young leaves by using modified Doyle and Doyle method [16].

#### 2.2.1. Isolation of RNA and 1st Strand cDNA Synthesis

Approximately 5 g of leaves was ground to fine powder in liquid nitrogen. The powdered tissue was transferred into a prewarmed (60°C) 10 mL extraction buffer [2% cetyl trimethylammonium bromide (CTAB), 2% polyvinylpyrrolidone (PVP 40), 100 mM Tris HCl (pH 8.0), 25 mM ethylenediaminetetraacetic acid (EDTA), 2.0 M NaCl, and 2%  *β*-mercaptoethanol (added just before use)]. The mixture was incubated for 30 minutes and proceeded with addition of an equal volume of chloroform : isoamylalcohol [24 : 1 (*v*/*v*)]. The mixture was then vortexed for 1 minute and centrifuged at 8000 rpm for 30 min at 4°C. The upper aqueous phase was transferred to a 1.5 mL microcentrifuge tube and reextracted with equal volume of chloroform : isoamylalcohol. Next, one-third volume of 8 M lithium chloride solution was added to the upper aqueous phase in a new tube to a final concentration of 2 M. The tube was then incubated overnight at −20°C and centrifuged at 13,000 rpm for 30 min at 4°C. The supernatant was discarded and the pellet was washed with 500 *μ*L of 70% ethanol followed by 500 *μ*L of 80% ethanol. The pellet was air-dried and resuspended in 35 *μ*L DEPC-treated water. Finally, RNA was kept at −80°C for long-term storage.

First strand cDNA synthesis was carried out according to RevertAid First strand cDNA synthesis kit protocol (Fermentas). Approximately 1 *μ*g of total RNA isolated was treated with DNase I (Promega), to remove traces of genomic DNA. A 0.5 *μ*g of adaptor(dt)_17_ (5′-GACTCGAGTCGACATCGATTTTTTTTTTTTTTTTT-3′) primer was added to the purified RNA and made up to a final volume of 12.5 *μ*L using nuclease-free water. The mixture was incubated at 70°C for 5 min and chilled on ice. Then, 4 *μ*L of 5X RT-buffer (Fermentas), 2 *μ*L dNTPs (10 mM), and 0.5 *μ*L of 40 U/*μ*L RNase Inhibitor (Fermentas) were added and incubated at 37°C for 5 min. Lastly, 1 *μ*L of 200 U/*μ*L RevertAid M-MuLV RT (Fermentas) was added and incubated for 1 hour at 42°C. The reaction was then stopped by heating at 70°C for 10 minutes.

#### 2.2.2. Isolation of DNA and DNA Purification

Approximately 0.1 g of sample was ground in liquid nitrogen and mixed with prewarmed (65°C) 1 mL of CTAB extraction buffer (0.1 M Tris-HCl pH 8, 0.02 M EDTA pH 8, 1.4 M NaCl, 2% (*w*/*v*) CTAB powder, 1% (*w*/*v*) PVP, and 0.2%  *β*-mercaptoethanol) and incubated at 65°C for 1 hour. After incubation, the mixture was left for 5 min to cool to room temperature and mixed gently with 400 *μ*L of chloroform : isoamylalcohol (24 : 1). The tube was centrifuged at 13,000 rpm for 5 min at 4°C. The aqueous fraction was reextracted once with chloroform : isoamylalcohol (24 : 1). One volume of ice-cold isopropanol was added and DNA was precipitated overnight at −20°C. The next day, DNA was collected by centrifugation at 13,000 rpm for 15 min at 4°C. The pellet was washed with 1 mL of wash buffer (76% ethanol and 0.01 M ammonium acetate), air-dried and resuspended in 40 *μ*L TE buffer (10 mM Tris HCl pH 8.0 and 1 mM EDTA, pH 8.0).

Approximately 50 *μ*L of the isolated genomic DNA (*≈*15 *μ*g) was mixed with 0.1 *μ*L RNase A (10 mg/mL) and incubated at 37°C for 1 hour. Then, equal volume of P : C : I was added and the mixture was centrifuged at 13,000 rpm for 15 min at 4°C. The aqueous phase was transferred to a new tube. Next, 2/3 volume of isopropanol and 0.1 volume of 3 M NaOAc (pH 5.2) was added to the solution and left to precipitate overnight at −20°C. After centrifugation at 13,000 rpm for 15 min at 4°C, the pellet was washed with 1 mL wash buffer, air-dried, and dissolved in 50 *μ*L of TE buffer. 

### 2.3. RACE Primer Design

Initially *Adh* screening was done via RT-PCR using primers designed from *Washingtonia robusta*as described by Morton et al. [12]: morADH-f (5′-GGGTGCTGTAGGCCTTGC-3′) and morADH-r (5′-GATATCTGCATTTGAATGCG-3′). Subsequently, sago palm Adh-specific primers were designed (msadh-f: 5′-CTAGAGCTTCAGGGGCATCA-3′; msadh-r: 5′-TCAAACCTCTTGGGGTTCAC-3′) and used to isolate the cDNA via RACE. For the 5′RACE, another primer was designed from *Adh* sequences of *Elaeis guineensis *(GenBank Accession No. ACF06607) (5′-ATGGCAAGCACTGTTGGTCA-3′), denoted as egadh-f.

### 2.4. RACE Method

RACE was performed as described by Frohman et al. [19] with minor modifications. The 3′ RACE was conducted in a final volume of 25 *μ*L containing 3 *μ*L cDNA template, 1 X PCR buffer (Fermentas), 1.5 mM MgCl_2_ (Fermentas), 0.2 mM dNTPs (Fermentas), 0.4 *μ*M msadh-f (1st BASE), 0.4 *μ*M adaptor(dt)_17_primer (1st BASE), 1.25 U* Taq* polymerase (Fermentas), and 15.25 *μ*L nuclease-free water. PCR program parameters were: 94°C for 2 min, 35 cycles of 94°C for 45 sec, 65°C for 45 sec, 72°C for 1 min, and 72°C for 5 min. A 5′RACE was performed using egadh-f primer (5′ATGGCAAGCACTGTTGGTCA-3′) and PCR amplified with msadh-r. It was conducted in a final volume of 25 *μ*L containing 3 *μ*L cDNA template, 1X PCR buffer (Fermentas), 1.5 mM MgCl_2_ (Fermentas), 0.2 mM dNTPs (Fermentas), 0.4 *μ*M RACE2 (1st BASE), 0.4 *μ*M GSP3 (1st BASE), and 1.25 U* Taq* polymerase (Fermentas). PCR conditions were 94°C for 2 min, 35 cycles of 94°C for 30 sec, 60°C for 30 sec, 72°C for 45 sec, and 72°C for 5 min.

### 2.5. Full-Length *Adh* Amplification

**Figure 1:**
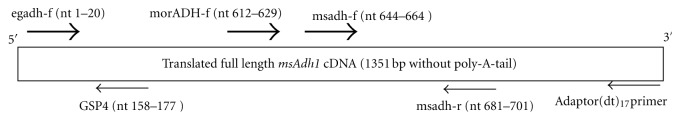
The orientation and position of primers used to isolate full length Adh cDNA.

Full length *Adh* cDNA amplification was carried out by mixing 2.5 *μ*L 10X HotStart buffer (Fermentas), 1.5 *μ*L 25 mM MgCl_2_ (Fermentas), 0.5 *μ*L 10 mM dNTPs (Fermentas), 1.0 *μ*L each for primer 10 *μ*M egadh-f (1st BASE) and 10 *μ*M adaptor(dt)_17_ (1st BASE), respectively, 1.25 U HotStart *Taq* DNA polymerase (Fermentas), 5.0 *μ*L first strand cDNA, and 13.25 *μ*L deionized water. The mixture was run under this condition: 95°C 4 min for 1 cycle, 95°C 45 sec, 60°C 1 min, 72°C 1.5 min for 35 cycles, and final extension for 5 min at 72°C.  Figure 1 shows the RACE strategy employed to isolate the full length Adh cDNA.

### 2.6. Genomic Walking

Genomic walking was carried out using a modified DNA walking method described by Ashoub and Abdalla [20]. Approximately 1.65 *μ*g of DNA was digested with 10 U of *Kpn*I, *Pst*I, and *Sac*I individually in a final volume of 50 *μ*L at 37°C overnight. The *Kpn*I and *Sac*I enzymes were heat-inactivated at 65°C while *Pst*I was heat-inactivated at 80°C for 20 minutes. Ten microlitres of the digested DNA was mixed with 3 U T4 DNA ligase (Fermentas), 1X ligation buffer (Fermentas), and 10 pmol of corresponding overhanging primers (Table 1) in a final volume of 20 *μ*L. The reaction was incubated at 16°C for 2 hours and then 4°C overnight.

The first round PCR was carried out in 25 *μ*L of reaction mixture containing 1X PCR buffer, 2 mM MgCl_2_, 200 *μ*M of each dNTPs, 10 pmol of each primer AP and GSP4, 1.25 U *Taq* polymerase (Fermentas), and 1 *μ*L of adaptor-ligated genomic DNA. The GSP4 primer was designed from the sequence derived from RACE and located 153 bp downstream from the *msAdh*1 start codon. Thermal cycling condition was set as predenaturing at 94°C for 3 min, denaturing at 94°C for 45 sec, annealing at 54–58°C for 45 sec, extension at 72°C for 1 min, repeat for 34 cycles, and a final extension at 72°C for 5 min. For nested PCR, 1 *μ*L of PCR product from the first round of PCR was used as the template and mixed with 1 X PCR buffer, 2 mM MgCl_2_, 0.2 mM dNTP, 0.4 *μ*M NP, and 0.4 *μ*M GSP4. The reaction mix was made up to 25 *μ*L with nuclease-free water. The PCR program was the same as indicated above. PCR products were analyzed on a 1.5% agarose gel and fragments detected were purified using GF-1 Gel DNA Recovery kit (Vivantis). Subsequently, the purified PCR products were cloned into pGEM-T easy vector and positive clones were sequenced. 

### 2.7. DNA Sequencing and Data Analysis

All PCR products were cloned into pGEM-T vector and sequenced. Several softwares were used to analyse the sequences such as the GenScan software [18] that was used to predict the protein coding region and intron region. The promoter region and transcription start site were predicted using the program Promoter Prediction [17]. Meanwhile, the amino acid sequences were predicted using an ORF detection program available from National Center for Biotechnology Information (NCBI). Alignment of protein sequences between ADH protein of sago palm with Adh from animal, bacteria, and other plants species derived from the GenBank database was done using European Bioinformatics Institute's (EBI) ClustalW multiple alignment software. The phylogenetic tree was produced to analyse sequence divergence using the Lasergene MegAlign program (DNASTAR Sequence Analysis Package, version 7.1.0) with the Clustal alignment algorithm.

## 3. Results and Discussion

### 3.1. RACE

**Figure 2:**
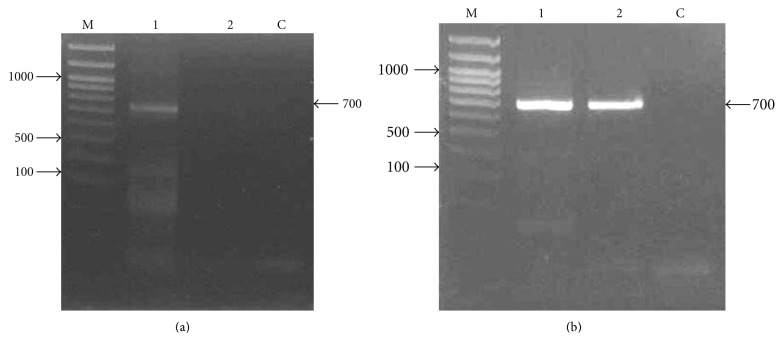
Agarose gel electrophoresis of RACE PCR products. (a) Gel electrophoresis picture of 3′RACE PCR product of young leaves on a 1.5% agarose gel. Lane 1 is the 3′RACE product and lane 2 is the PCR product with no RT template. (b) Gel electrophoresis picture of PCR product using primer egadh-f and msadh-r on 1.5% agarose gel. Lanes 1 and 2 are the 5′RACE-PCR. Lane C is the control and lane M contained the Forever 100 bp DNA ladder (Seegene). Visible fragment showing the 700 bp fragment.

RNA isolated from leaf samples were analysed on 1.0% agarose gel-stained with ethidium bromide. Two distinct RNA fragments, the 28S and 18S rRNA, were clearly observed (data not shown) which showed no apparent RNA degradation. The total RNA extract were then treated with RQ1 RNase-Free DNase (Promega) to remove trace amount of genomics DNA from the total RNA. The mRNA was converted to cDNA using adaptor(dt)_17_primer and subsequently used in the RACE. Initially several PCRs were conducted using primers designed from *W. robusta*. These PCR produced several *Adh*-specific amplicons (data not shown) which were isolated and sequenced. From these results, the primers used in RACE were designed and used in the 3′- and 5′RACE. The 3′RACE was conducted using msadh-f and adaptor(dt)_17_ and at the annealing temperature of 65°C. A fragment of approximately 700 bp was detected, isolated, and sequenced (Figure 2(a)). 

Meanwhile, the forward primer used for the 5′RACE (egadh-f) was designed based on the *Adh* sequence of oil palm that have showed a high-sequence similarity with the 3′RACE sequence (data not shown). Thus, the 5′RACE was performed using egadh-f primer along with sago palm *Adh*-specific primer. The amplification produced a fragment of approximately 700 bp fragment (Figure 2(b)) that was then isolated and sequenced. Subsequently the assembly of the 5′- and 3′RACE contigs showed overlapping region (58 bp) to produce a complete *Adh *cDNA. A complete *Adh* cDNA was then amplified using the primers egadh-f and adaptor(dt)_17_. Approximately 1.3 kb fragment was obtained and sequenced that confirm the nucleotide sequence from the two assembled contigs. Sequence search via BLAST against the nucleotide database in Genebank NCBI showed a high degree of similarity with *Adh*1 from different plant species.

### 3.2. Genomic Walking

**Figure 3:**
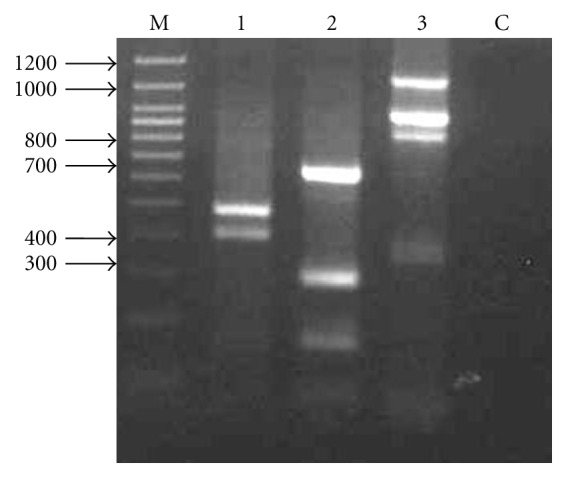
Agarose gel electrophoresis of genomic walking nested PCR products of young leaves. The restriction enzymes used for DNA digestion were indicated above the gel photo. Lanes 1, 2, and 3 contained the PCR products using *Kpn*I, *Pst*I, and *Sac*I, respectively. Lane M contained the 1 Kb ladder (Fermentas) and C is negative control.

The genomic walking was performed with two aims; first was to verify the translation start site sequence of sago palm *msAdh*1 cDNA since the egadh-f primer sequence used in 5′RACE was designed from oil palm. The second aim was to isolate the regulatory sequences of *msAdh*1. The nested PCR of genomic walking produced two fragments with the sizes of 400 bp (YKpn400) and 500 bp (YKpn500) when restriction enzyme of *Kpn*I was used. For the *Pst*I-restricted genomic DNA, the nested PCR produced three fragments; two clear fragments at approximately 700 bp (YPst700) and 300 bp (YPst300) and one faint fragment at approximately 200 bp. Lastly, genomic walking using *Sac*I-restricted genomic samples produced three clear fragments at around 1.2 kb (YSac1200), 1 kb (YSac1000), and 800 bp (YSac800) and one faint fragment at around 350 bp (YSac300) (Figure 3). 

**Figure 4:**
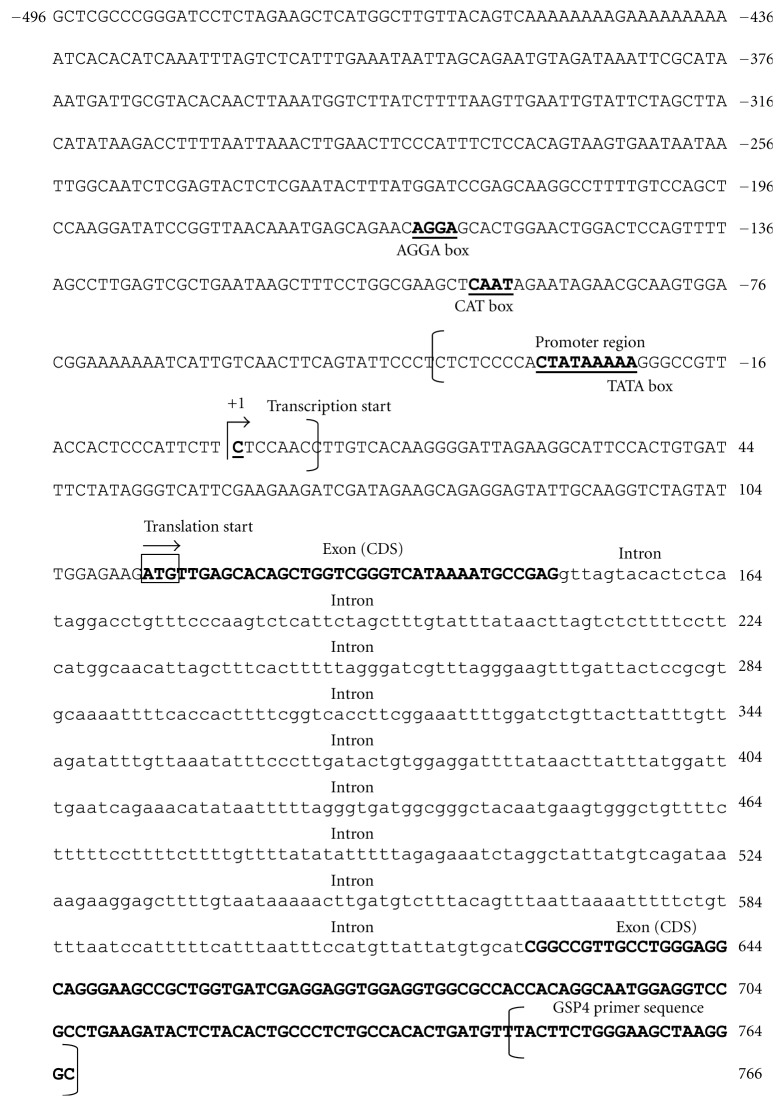
Analysis of the upstream sequences of *msAdh*1 gene. A putative promoter region (in parenthesis) is identified with the detection of the transcription and translation start sites using *Promoter Prediction* [17]. The putative transcription start site is designated as +1 (indicated by an arrow) and translation start site is shown in box. The intron region is indicated by small caps, meanwhile the protein coding region (bold) was predicted by using GenScan software [18]. The putative TATA box (TATAAAAA), CAT box (CAAT), and AGGA box are indicated in bold and underlined.

The fragments produced from nested PCR were excised and sequenced. Sequencing results obtained were analysed using BLAST against NCBI genebank nonredundant nucleotide collection database. BLASTX search showed that the three sequences (YPst300, YSac1200, and YKpn400) matched with *Adh* gene. The 1.2 kb fragment (YSac1200) was selected for further analysis of the protein coding sequences (CDS) using GenScan software [18] (Figure 4). The analysis predicted the presence of an intron and splicing sites that were deduced according to the GT/AG of the intron/exon junction [21–23]. In addition, the predicted intron in sago palm *msAdh*1 is rich in AT base pair (69%) which is similar to introns found in other species [24]. Since the consensus sequence at both the GT/AG boundary almost identical to the sequence at the predicted intron site, it is likely that it is the first intron site of *msAdh*1 gene. After removing the intron sites and realignment with the *msAdh*1 cDNA, a 70% sequence similarity of was found between the 5′end in both genomic DNA and cDNA of *msAdh*1.

Promoter region and transcription start site were also predicted from the isolated sequence using the program Promoter Prediction [17]. From the predicted transcription start site, a putative TATA box with the sequence CTATAAAAA was found at the positions −31 bp and −23 bp from transcription start site. The sequence and location closely corresponds to the plant TATA box consensus sequence (C/G)TATA(T/A)(A1–3)(C/T)A [25, 26]. Furthermore, Breathnach and Chambon [21] and Lin et al. [25] suggested that the distance between putative TATA box and transcription start site in most genes are between 25–30 bp or 32 ± 7 bp, respectively. The CA dinucleotide that is a typical initiation site for eukaryotes class II genes usually found at region −1 to +1 [23, 27] was also found in this study, however, the CA-dinucleotide was located −6 from transcription start site. In addition, putative CAT and AGGA box [26, 28] that may play a role in promoter efficiency were also found near the TATA box at position −98 bp to −95 bp and −162 bp to −159 bp, respectively, (Figure 4). All these elements found in the promoter region are necessary for accurate initiation of basal transcription.

### 3.3. Analysis of the Predicted Protein Sequence of Sago Palm Adh1

**Figure 5:**
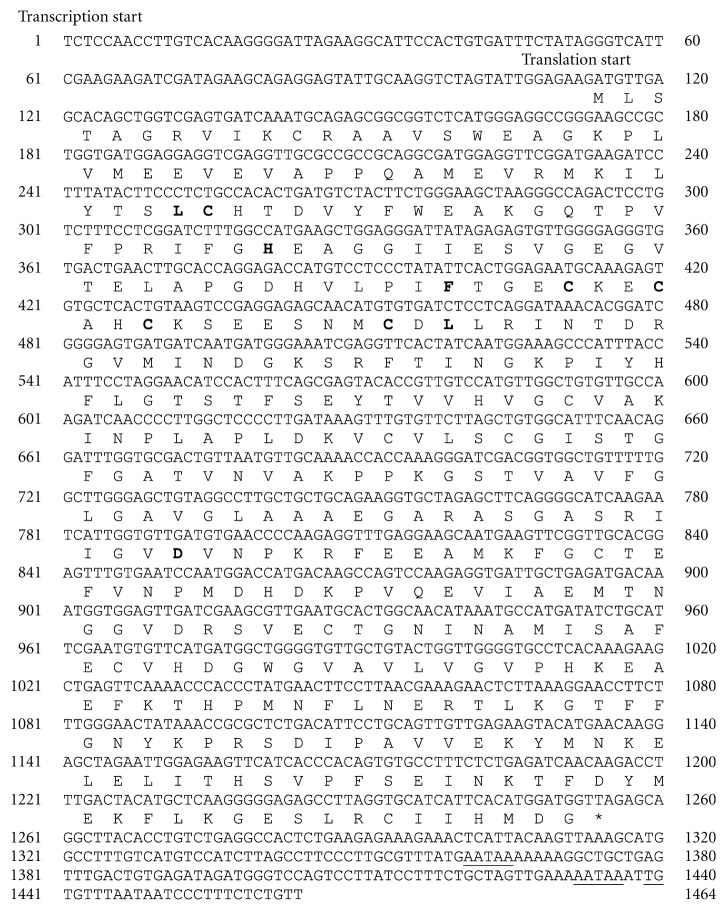
Nucleotide *msAdh*1 sequence of sago palm leaf cDNA and deduced amino acid sequence. Underlined regions represent putative polyadenylation sites (AATAAA) and terminal transcription factor (TGTGTTTA). The conserved amino acids of Adh are indicated in bold.

The *msAdh*1 cDNA sequence was determined to be 1464 bp in length with 1143 bp open reading frame encoding the Adh protein, 113 bp of 5′ untranslated region, and a 208 bp 3′ untranslated region not including the poly(A) tail (Figure 5). Further *in silico* sequence analysis showed that the nucleotide encodes for 380 amino acids. Based on the cDNA sequence data, the predicted molecular mass of msAdh1 was 41.8 kDa. At the 3′ terminus of the *msAdh*1 cDNA sequence (Figure 5), two polyadenylation signals (AATAAA) conserved in plants [29] were located at positions 1218 bp and 1319 bp, respectively. The sequence TGTGTTTA that is homologous to terminal transcription factor consensus [30] was also found between positions 1326 to 1333.

**Figure 6:**
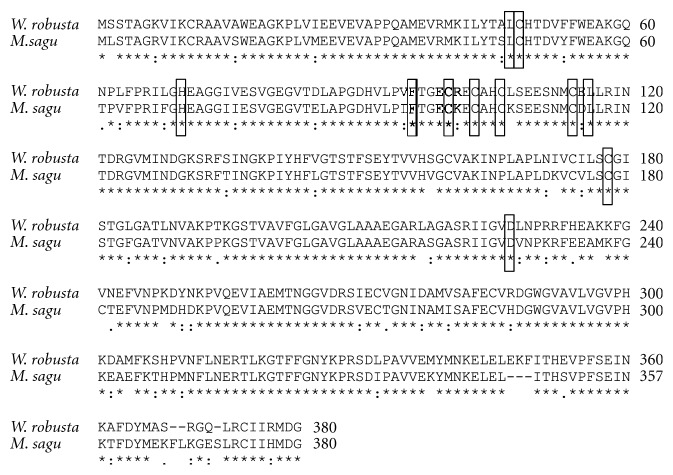
Alignment of alcohol dehydrogenase amino acid sequence between *W. robusta* and *M. sagu*. The boxes showed the conserved regions (Cys-48/His-70/Cys-178, Cys-100/Cys-103/Cys-106/Cys-114, Asp-227, Phe-93, Leu-47, and Leu-116) in the amino acid sequences. Dash is included in the sequences to maximize the homology.

The msAdh1 sequence was further analysed for motifs and factors specific for *Adh* regulation. Conserved amino acids of two zinc-binding domains found in oil palm and *W. robusta *located at Cys-48/His-70/Cys-178 and Cys-100/Cys-103/Cys-106/Cys-114 [12], respectively, were also present and conserved in sago palm msAdh1 (Figure 6). Other motifs include the Asp-227 that binds to the adenosine ribose of the coenzyme and the amino acids Phe-93, Leu-57, and Leu-116 that bind to alcohol substrate [31], were also found with exception that Leu-57 was found at location 47 for both oil palm and *W. robusta* (Figure 6). 

### 3.4. Similarity Analysis of Sago Palm *msAdh*1 with Other Species

Sequence homology search of deduced *msAdh*1 amino acid sequence using NCBI BLASTX showed 91% identity with the oil palm (*E. guineensis*) deduced Adh amino acid sequence. Comparisons were also conducted with amino acid sequences of other selected organisms (Table 2). The msAdh1 of sago palm showed highest identity (91%) with oil palm Adh. This was followed by 87% identity to rice and maize Adh1, 85% identity to *W. robusta* AdhB, and 82% identity to Adh from *Arabidopsis*. 

**Figure 7:**
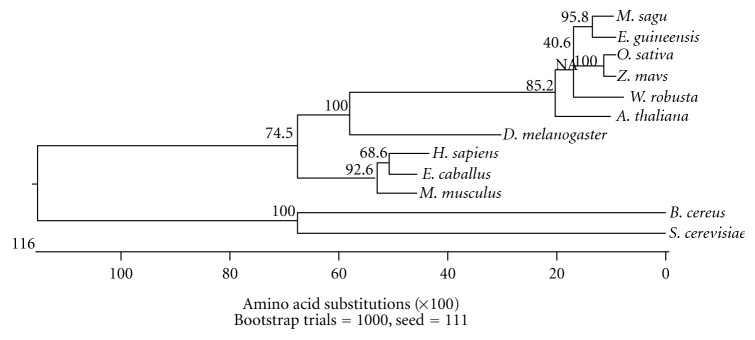
Phylogenetic tree of msAdh1 of sago palm and other species Adh. Tree was constructed by using clustal method of Lasergene Megalign (DNASTAR, Inc., Madison, WI) based on amino acid similarities. Numbers represent bootstrap values. Scale bar indicates levels of sequence divergence.

The percentage of divergence was calculated by comparing sequence pairs in relation to the phylogeny reconstructed by MEGALIGN. The percent divergences of *M. sagu* from *E. guineensis* and *W. robusta *were determined to be 8.7% and 15.8%, respectively. In contrast, both microorganisms (yeast and *Bacillus cereus*) had higher percentages of divergence from sago palm. From the phylogenetic tree (Figure 7), animals, plants, and microorganism are classified into three distinct groups. In plants, monocots and dicots Adhs form two distinct groups. The Adh1 amino acid sequence between animals and plants is closely related compared to microorganisms. As expected, the sago palm *msAdh*1 is similar to other plant forms and in particular, closer to the monocot compared to dicots. Furthermore, the result suggested that sago palm *msAdh*1 is more likely related to oil palm than to rice and maize, and shares a common *Adh* ancestor. 

## 4. Conclusion

*Adh* cDNA from sago palm have been successfully isolated using a combination of RACE and genomic walking method. Analysis of nucleotide sequence and predicted amino acid indicated that the sago palm cDNA is *Adh1*. The full length of *msAdh*1 cDNA was determined to be 1464 bp containing the 5′ and 3′ untranslated regions and a deduced amino acid of 380. The regulatory region of basal *msAdh*1 was isolated and found to contain the promoter sequences and conserved motifs corresponding to Adh regulation such as the two zinc-binding domains, binding domains to adenosine ribose of the coenzyme, and alcohol substrate.

## Figures and Tables

**Table 1:** Nucleotide sequence of primers used in genomic walking. Overhanging primers (OHP) with *Kpn*I, *Pst*I, and *Sac*I overhang sequence indicated in italic bold adaptor primer (AP), nested primer (NP), and *Adh* gene specific primer (GSP4).

Primer name	Sequence
OHP *Kpn*I	5′-GAATTCGAGCTCGCCCGGGATCCTCTAGA***GTAC ***-3′
OHP *Pst*I	5′-GAATTCGAGCTCGCCCGGGATCCTCTAGA***TGCA ***-3′
OHP *Sac*I	5′-GAATTCGAGCTCGCCCGGGATCCTCTAGA***AGCT ***-3′
AP	5′-GAATTCGAGCTCGCCCGGGAT-3′
NP	5′-GCTCGCCCGGGATCCTCTAGA-3′
GSP4	5′-GCCCTTAGCTTCCCAGAAGT-3′

**Table 2:** Comparison between msAdh1 with other species Adh deduced amino acid sequences.

Species	Protein	Length of deduced amino acid	Amino acid identity (%)	Accession number
*Elaeis guineensis*	Adh	380	91	ACF06607
*Washingtonia robusta*	AdhB	380	85	AAB39597
*Zea mays*	Adh1	379	87	CAA27681
*Oryza sativa *	Adh1	379	87	Q75ZX4
*Arabidopsis thaliana *	Adh	379	82	AAL90991
*Drosophila melanogaster*	Adh	379	49	AAB02520
*Mus musculus*	Adh1	375	48	NP_031435
*Homo sapiens*	Adh1	375	49	AAC41757
*Equus caballus*	Adh	374	51	1MG0_A
*Saccharomyces cerevisiae*	Adh1	347	18	2HCY_A
*Bacillus cereus*	Adh1	345	15	ZP_04323237
